# Personalized delayed anticoagulation therapy alleviates postoperative bleeding in total knee arthroplasty (TKA) patients

**DOI:** 10.1002/jeo2.70074

**Published:** 2024-10-30

**Authors:** Xuefeng Luo, Dehua Wang, Wei Xu, Jing Zou, Runxing Kang, Tao Zhang, Xi Liang, Junyi Liao, Wei Huang

**Affiliations:** ^1^ Department of Orthopaedic Surgery The First Affiliated Hospital of Chongqing Medical University Chongqing China; ^2^ Chongqing Municipal Health Commission Key Laboratory of Musculoskeletal Regeneration and Translational Medicine Orthopaedic Research Laboratory of Chongqing Medical University Chongqing China

**Keywords:** ecchymosis, hyperfibrinolysis, personalized delayed anticoagulation, perioperative bleeding, total knee arthroplasty

## Abstract

**Purpose:**

Ecchymosis is one of the most common complications following total knee arthroplasty (TKA), which is closely related to postoperative bleeding. However, it is still controversial whether anticoagulation treatment should be continued for postoperative ecchymosis patients. We suppose that personalized delayed anticoagulation therapy could be beneficial for decreasing postoperative bleeding.

**Methods:**

A total of 201 TKA patients were retrospectively included in this study, among whom ecchymosis patients received drug anticoagulation treatment 1–2 days later than usual, while nonecchymosis patients received regular drug anticoagulation treatment. The perioperative blood loss, coagulation state, fibrinolytic state and complications were collected and analyzed.

**Results:**

Eighty‐nine patients (44.3%) developed ecchymosis within 3 days after TKA. There were no differences in baseline characteristics between the two groups. In the ecchymosis group, higher K values and lower calculated coagulation index values were observed in thromboelastography, along with greater total blood loss and a more significant decrease in haemoglobin levels on postoperative Day 1 (POD1) compared to the nonecchymosis group. Additionally, the ecchymosis patients exhibited higher levels of fibrinogen degradation products and D‐dimer (D‐D) on POD1, with no differences noted on POD3, indicating that patients with ecchymosis are in a relatively hypocoagulable and hyperfibrinolytic state compared to those without ecchymosis. Therefore, the delayed anticoagulation treatment proved beneficial for correcting these postoperative conditions. No statistically significant differences were found between the two groups in postoperative complications, demonstrating that delayed anticoagulation treatment is safe.

**Conclusion:**

Patients with ecchymosis exhibited a relatively hypocoagulable and hyperfibrinolytic state with a stronger tendency for postoperative bleeding. Delayed anticoagulation in ecchymosis patients could effectively prevent further exacerbation of postoperative bleeding by avoiding sustained hypocoagulable and hyperfibrinolysis states. Personalized delayed anticoagulation therapy could be beneficial for managing postoperative ecchymosis for TKA patients.

**Level of Evidence:**

Level IV.

**Abbreviations**
ALT: alanine aminotransferase
APPT: activated partial thromboplastin time
AST: aspartate aminotransferase
BMI: body mass index
CI: coagulation index
COPD: chronic obstructive pulmonary disease
D‐D: D‐dimer
DVT: deep vein thrombosis
EPL: estimated percent lysis
FDP: fibrinogen degradation products
Hb: haemoglobin
INR: international normalized ratio
IVT: intramuscular vein thrombosis
LY30: percentage of clot which has lysed after 30 min
MA: maximum amplitude
PE: pulmonary embolism
POD1: postoperative Day 1
PT: prothrombin time
PTA: prothrombin time activity
PTR: posttransfusion refractoriness
TBL: total blood loss
TEG: thromboelastography
TKA: total knee arthroplasty
TT: thrombin time
TXA: tranexamic acid
VTE: venous thromboembolism

## INTRODUCTION

Total knee arthroplasty (TKA) stands out as one of the most effective surgeries for treating end‐stage knee joint diseases [17, 19, 23]. Concurrent with the rapid rise in the number of TKA, the concerns for postoperative complications, including venous thromboembolism (VTE), infection and chronic pain and so on continue to grow [9, 13, 15].

VTE, which encompasses deep vein thrombosis (DVT) and pulmonary embolism (PE), is one of the most common and highest‐risk complications in large‐scale orthopaedic operations, with an incidence rate ranging from 40% to 60% [31]. Up to 70% of VTE cases have no symptoms and resolve within a month, about 6% identified DVT cases and 12% of PE cases end in death [22]. Based on these facts, prophylactic treatment of VTE is widely accepted worldwide, leading to a decrease in VTE cases after TKA to 0.5%–1% [7]. However, alongside the widespread and standardized use of anticoagulation treatment, there has been an increasing trend in postoperative bleeding and wound complications [1, 27].

Among the bleeding‐related complications, perioperative ecchymosis around the surgical site is often accompanied by severe swelling, pain, local infection and mental stress, which can prolong the recovery time after TKA [12, 20]. Recent studies characterized that postoperative ecchymosis could be easily observed and served as a ‘barometer’ for bleeding and hypercoagulable states [1, 26]. While the absence of anticoagulants may put patients at risk of VTE, excessive anticoagulant therapy can increase the risk of bleeding [20, 26].

Given these insights, we assumed that patients after TKA exhibit different bleeding risks and coagulation states, and those with ecchymosis after TKA are in a relatively hypocoagulable state, leading to a more pronounced tendency for bleeding. Delayed anticoagulation therapy for ecchymosis patients after TKA could decrease the excessive bleeding risk without increasing the VTE risk. Thus, the present study analyzed perioperative blood loss, coagulation state, fibrinolytic state and complications between ecchymosis patients receiving delayed anticoagulation therapy and nonecchymosis patients with routine anticoagulation therapy after TKA.

## MATERIALS AND METHODS

### Study design

This retrospective study was approved by the local ethics committee (Approval No. K2023‐636) and was registered in the Chinese Clinical Trial Registry (Registration No. ChiCTR2400079496) under the title ‘Study on the safety and effectiveness of personalized anticoagulation in reducing perioperative bleeding after joint replacement’ (https://www.chictr.org.cn/showproj.html?proj=215999). Patients who underwent TKA in the orthopaedic department from July 2021 to December 2023 were retrospectively analyzed in this study.

### Eligibility criteria

To mitigate potential heterogeneity among patients, strict inclusion and exclusion criteria were implemented. The specific criteria are presented in Table 1 for reference.

**Table 1:** The inclusion and exclusion criteria.

Inclusion criteria	Exclusion criteria
1. Received unilateral primary TKA due to end‐stage knee diseases.	1. Combined with severe cardiovascular or cerebrovascular diseases (uncontrolled coronary heart disease, acute myocardial infarction, cerebral infarction, intracranial aneurysm etc.).
2. Age ≥18 years.	2. Combined with obvious bleeding tendencies (History of gastrointestinal and cerebral haemorrhage, purpura, etc.).
	3. Combined with systemic or local venous thromboembolism.
	4. Combined with coagulation disorders or a history of anticoagulant therapy (coagulation disorders are abnormal manifestations caused by vascular wall abnormalities, lack of coagulation factors, thrombocytopenia, drug factors, etc.).
	5. Combined with significant hepatic or renal dysfunction (ALT > 200 U/L, AST > 200 U/L, glomerular filtration rate <60 mL/min and the serum creatinine >178 µmol/L).
	6. Patients who underwent bilateral or revision TKA.
	7. Patients with incomplete medical records.

Abbreviations: ALT, alanine aminotransferase; AST, aspartate aminotransferase; TKA, total knee arthroplasty.

### Perioperative management

Every patient underwent standard surgery and received perioperative care for elective TKA. All surgeries were conducted by a consistent team comprising two senior surgeons, with patients in the supine positions and accepting general anaesthesia. A pneumatic tourniquet with a constant pressure of 30 kpa was consistently utilized during the entire surgical procedure. Before making the skin incision, a tourniquet and tranexamic acid (TXA) (10 mg/kg, intravenous) were administered. TXA (1 g) was applied topically to the exposed joint and wound sites via a syringe. Following deep suturing, wounds were closed using standard subcutaneous and intradermal sutures, and no drainage devices were employed. The anaesthetist documented intraoperative blood loss, including blood collected in suction canisters. No blood transfusions were administered during the surgical procedures.

For DVT prevention, from the first postoperative day (POD), patients received a subcutaneous injection of 4000 IU of low‐molecular‐weight‐heparin for anticoagulation therapy. Physical prevention of VTE, such as ankle pump training, intermittent pneumatic compression and so on, was also applied for all TKA patients.

After the surgery, patients were monitored closely for the ecchymosis manifestation and then categorized into two groups: those with ecchymosis and those without ecchymosis. Upon confirmation of ecchymosis, the routine administration of 4000 IU of low‐molecular‐weight heparin was delayed. The area of ecchymosis was evaluated by the researcher using the patient's own hand, with one hand corresponding to 1% of the total body surface area. This assessment was accurately recorded in the medical records.

Each patient had bilateral lower extremity venous ultrasonography on both the day before the surgery and the third day after the surgery. Additionally, blood tests were conducted on the day before the surgery as well as on the 1st and 3rd days after the surgery. These blood tests included a complete blood count, coagulation profile and so on. Simultaneously, thromboelastography (TEG) was applied for detecting coagulation function. The TEG test was conducted using a computerized TEG coagulation analyzer (TEG Model 5000; Haemoscope Corporation). All thermoelectric generator statistics were documented, such as R‐time, K‐time, maximum amplitude, α angle and calculated coagulation index (CI). R‐time refers to the duration needed to produce a clot stiffness of 2 mm amplitude, which signifies the beginning of clot formation. This measurement is associated with the functioning of enzymatic clotting factors [24]. K‐time refers to the duration between the end of R and the creation of a 20 mm clot, whereas α angle indicates the steepness of the TEG trace at R, offering comparable information to K‐time. Both measures demonstrated the speed at which fibrin accumulates and forms connections and are regarded as indicators of fibrinogen functionality. The greatest amplitude corresponds to the highest intensity of the clot and is determined by the quantity and performance of platelets [11]. The Nadler formula was employed to calculate the total amount of blood loss: Patient blood volume = k1 × height^3^ (m) + k2 × weight (kg) + k3, where for males, k1 = 0.3669, k2 = 0.03219 and k3 = 0.6041, and for females, k1 = 0.3561, k2 = 0.03308 and k3 = 0.1833. Total blood loss (TBL) = Patient blood volume × (Hct preop − Hct postop). To determine the total amount of blood lost, multiply the patient's blood volume by the difference between the haematocrit levels before and after the operation.

### Data analyses

The statistical analysis was conducted using the SPSS 24.0 (SPSS Inc.) programme. A Kolmogorov–Smirnov test was conducted to ascertain if the data adhered to a normal distribution. In the context of a normal distribution, numerical data was briefly presented by employing the measures of mean and standard deviation. The independent *t* test was employed to identify disparities in normally distributed numerical data, while the Mann–Whitney U test was utilized for nonnormally distributed numerical values. A *p *< 0.05 was deemed to be statistically significant.

## RESULTS

### Patient information and baseline characteristics

**Figure 1:**
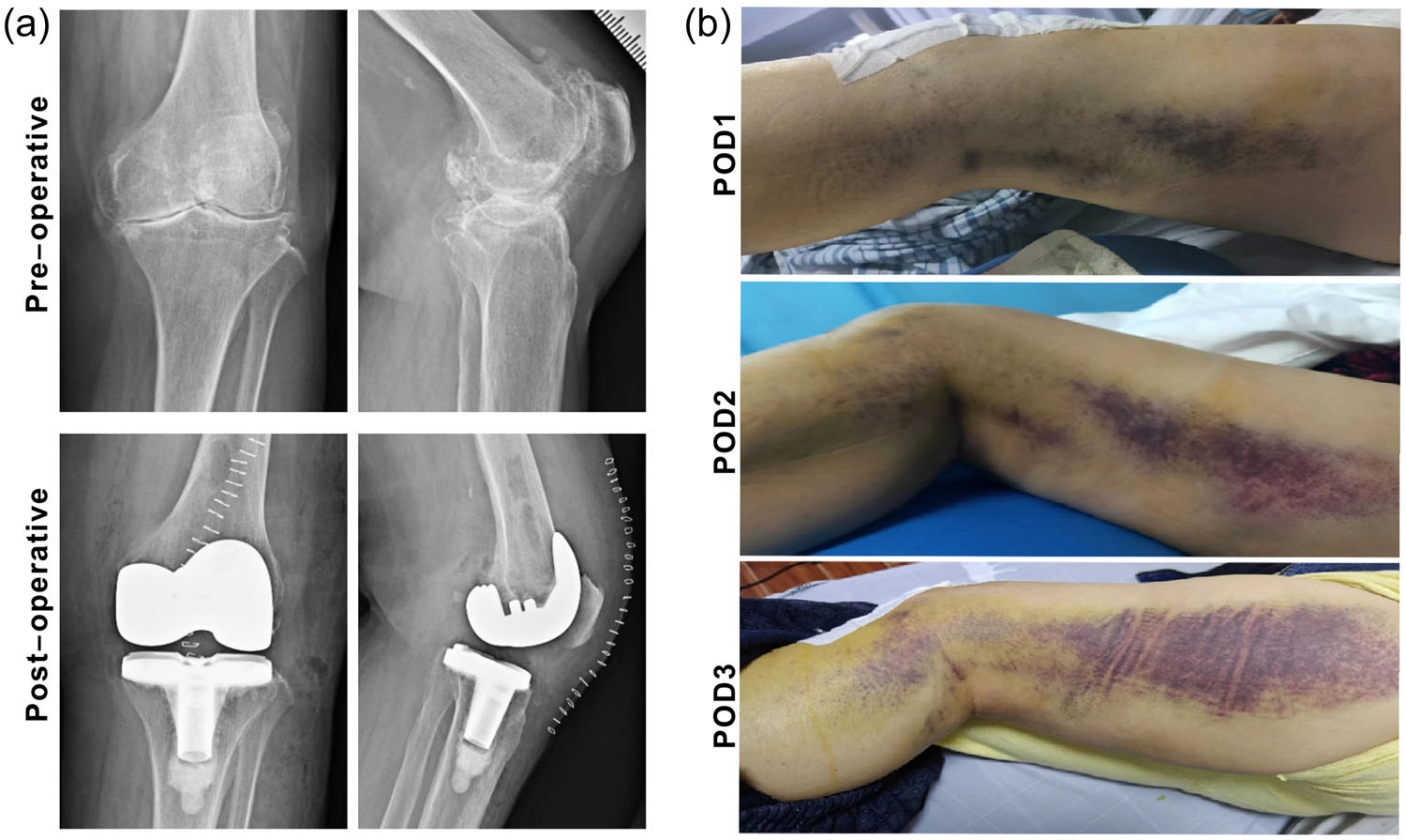
Ecchymosis after total knee arthroplasty. (a) Preoperative and postoperative X‐ray were exhibited. (b) Ecchymosis change from postoperative Day 1 to postoperative Day 3.

Patients with post‐TKA ecchymosis are at high risk of progressive increase in the area of ecchymosis. One patient with typical ecchymosis is shown in Figure 1. As anticoagulation therapy may aggravate ecchymosis, we proposed that delayed anticoagulation treatment would decrease the risk of postoperative exacerbation of ecchymosis.

**Figure 2:**
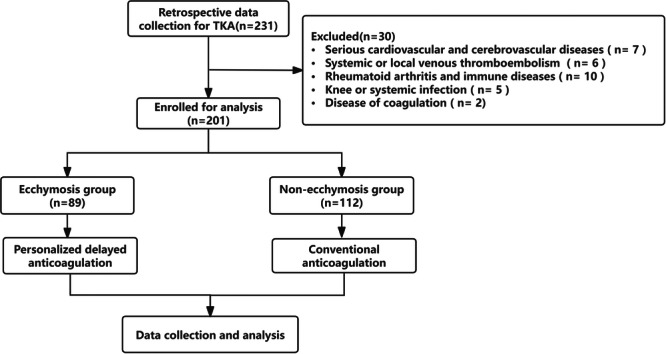
The flow chart of the study.

A cohort of 231 patients who underwent TKA from July 2021 to December 2023 were assessed for eligibility, and 201 patients, including 171 females and 30 males, met the criteria for inclusion in this study. A total of 89 individuals, accounting for 44.3% of the sample, experienced different levels of perioperative ecchymosis within 3 days after TKA. Next, patients were divided into the ecchymosis group (*n *= 89) and the nonecchymosis group (*n* = 112) (Figure 2). Demographic characteristics, haematologic results and preoperative comorbidities were shown in Table 2, and no statistically significant difference was found between the ecchymosis and nonecchymosis groups.

**Table 2:** Demographic, haematologic results and preoperative comorbidities in the ecchymosis and nonecchymosis groups.

Variables	Nonecchymosis (*n* = 112)	Ecchymosis (*n* = 89)	t or *χ* ^2^	*p* Value
Age (year)	68.83 ± 7.36	69.36 ± 7.08	−0.515	0.607
Male, *n* (%)	18 (16.1)	12 (13.5)	0.262	0.609
Female, *n* (%)	94 (83.9)	77 (86.5)		
Height (cm)	155.88 ± 8.15	156.12 ± 6.07	−0.248	0.805
Weight (kg)	63.65 ± 10.25	64.42 ± 10.23	−0.532	0.595
Prealbumin (g/dL)	214.75 ± 40.31	219.06 ± 37.44	−0.776	0.439
ALT (U/L)	18.57 ± 17.26	17.28 ± 13.09	0.584	0.56
AST (U/L)	21.19 ± 11.91	20.27 ± 9.17	0.599	0.55
Urea (mmol/L)	6.19 ± 1.96	6.26 ± 1.75	−0.278	0.781
BMI (kg/m^2^)	26.14 ± 3.4	26.42 ± 3.87	−0.545	0.587
BMI < 18, *n* (%)	0	0	0.491	0.782
18 ≤ BMI < 25, *n* (%)	41 (36.6)	30 (33.7)		
25 ≤ BMI < 30, *n* (%)	57 (50.9)	45 (50.6)		
30 ≤ BMI, *n* (%)	14 (12.5)	14 (15.7)		
Albumin (g/dL)	41.8 ± 4.21	42.4 ± 2.64	−1.176	0.241
38 ≤ Albumin, *n* (%)	106 (94.6)	85 (95.5)	0.078	0.78
Albumin < 38, *n* (%)	6 (5.4)	4 (4.5)		
Hb (g/L)	126.19 ± 12.52	126.89 ± 13.43	−0.381	0.703
110 < Hb, *n* (%)	103 (92.0)	78 (87.6)	1.229	0.541
100 < Hb ≤ 110, *n* (%)	8 (7.1)	9 (10.1)		
90 < Hb ≤ 100, *n* (%)	1 (0.9)	2 (2.3)		
80 < Hb ≤ 90, *n* (%)	0	0		
Hb ≤ 80, *n* (%)	0	0		
Comorbidities (no. [%])				
Hypertension	44 (39.3)	45 (50.6)	2.556	0.11
Diabetes	22 (19.6)	16 (18)	0.09	0.765
Fatty liver	20 (17.9)	25 (28.1)	2.988	0.084
Hyperlipidemia	5 (4.5)	5 (5.6)	0.14	0.709
Smoking	18 (16.1)	14 (15.7)	0.004	0.948
Regular alcohol use	30 (26.8)	28 (31.5)	0.528	0.467
COPD	3 (2.7)	3 (3.4)	0.082	0.775
Renal insufficiency	1 (0.9)	1 (1.1)	0.027	0.87

Abbreviations: ALT, alanine aminotransferase; AST, aspartate aminotransferase; BMI, body mass index; COPD, chronic obstructive pulmonary disease; Hb, haemoglobin.

### Preoperative coagulation status

Perioperative coagulation status change could be associated with ecchymosis formation [2, 14, 26, 28]. Hence, we first analyzed preoperative coagulation status. As shown in Table 3, it can be observed that there was no statistically significant difference between ecchymosis and nonecchymosis groups in terms of preoperative platelet count, coagulation profile and TEG parameters. These results indicated that the change in coagulation status after TKA could be more closely associated with postoperative ecchymosis formation.

**Table 3:** Preoperative coagulation parameters in the ecchymosis and nonecchymosis groups.

Variables	Nonecchymosis (*n* = 112)	Ecchymosis (*n* = 89)	t or *χ* ^2^	*p* Value
APTT (s)	34.70 ± 3.51	33.78 ± 4.96	1.552	0.122
PT (s)	12.85 ± 0.71	13.02 ± 0.73	−1.664	0.098
TT (s)	17.13 ± 1.17	18.26 ± 1.63	−1.113	0.267
INR	0.98 ± 0.06	1.38 ± 0.58	−1.044	0.299
PTA (%)	106.12 ± 11.21	104.30 ± 11.62	1.127	0.261
PTR	0.99 ± 0.05	1.00 ± 0.05	−0.887	0.376
Platelet (10^9^/L)	208.95 ± 59.14	208.29 ± 52.26	0.082	0.935
R (min)	6.44 ± 1.58	6.25 ± 1.41	0.893	0.373
K (min)	1.63 ± 0.69	1.68 ± 0.68	−0.546	0.585
α‐angle (°)	66.30 ± 6.67	66.09 ± 5.20	0.245	0.806
MA (mm)	64.12 ± 4.80	63.08 ± 4.09	1.169	0.107
CI	0.28 ± 1.96	0.25 ± 1.58	0.216	0.9
CI < −3, *n* (%)	3 (2.7)	4 (4.5)	0.622	0.733
−3 ≤ CI ≤ 3, *n* (%)	107 (95.5)	84 (94.4)		
3 < CI, *n* (%)	2 (1.8)	1 (1.1)		
LY30 (%)	0.5 (0, 1.2)	0.4 (0, 1.2)	−0.447	0.656
LY30 ≤ 3%, *n* (%)	105 (93.7)	82 (92.1)	0.2	0.655
3% < LY30, *n* (%)	7 (6.3)	7 (7.9)		

Abbreviations: APPT, activated partial thromboplastin time; CI, calculated coagulation index; EPL, estimated percent lysis; INR, international normalized ratio; K, K time; LY30, percentage of clot which has lysed after 30 min; MA, maximum amplitude; PT, prothrombin time; PTA, prothrombin time activity; PTR, posttransfusion refractoriness; R, reaction time; TEG, thromboelastography; TT, thrombin time.

### Surgical information and perioperative blood loss

As for operative information, we found no significant difference in the operation time, tourniquet time, intraoperative blood loss and patient blood volume between the two groups (Table 4). Patients in the ecchymosis group experienced more postoperative hospital stays (*p *< 0.001). As ecchymosis indicates postoperative bleeding, we analyzed blood loss postoperatively. On POD1 and POD3, there was no statistically significant disparity in mean haemoglobin levels between the two groups of patients (Table 5, *p* = 0.119 and 0.244). As for haemoglobin lower than 100 g//L and the change in haemoglobin levels, the ecchymosis group exhibited significantly greater haemoglobin level decreasing compared with the nonecchymosis group (Table 5, *p* = 0.02 and *p* = 0.003), and this difference in existence on POD3. As for the TBL postoperatively (Table 5), we found that the ecchymosis group exhibited more blood loss on POD1 (*p* = 0.009), and the difference was in existence on POD3 (*p* = 0.095). These results suggested that patients with ecchymosis possess a more obvious bleeding tendency on POD1, and this tendency disappeared with delayed anticoagulation treatment on POD3 (*p* = 0.074).

**Table 4:** Surgical information in the ecchymosis and nonecchymosis groups.

Variables	Nonecchymosis (*n* = 112)	Ecchymosis (*n* = 89)	t/z	*p* Value
Operation time (min)	92.29 ± 24.06	97.37 ± 29.54	−1.345	0.18
Tourniquet time (min)	74.34 ± 25.22	78.82 ± 29.03	−1.170	0.243
Intraoperative blood loss (mL)	41.24 ± 5.84	41.99 ± 5.14	−0.95	0.343
Patient blood volume (mL)	3713.93 ± 551.69	3737.11 ± 501.68	−0.308	0.759
Postoperative hospital stays (day)	3.24 ± 0.43	3.75 ± 0.70	−6.081	<0.0001

**Table 5:** Perioperative blood loss in ecchymosis and nonecchymosis groups.

Variables	Nonecchymosis (*n* = 112)	Ecchymosis (*n* = 89)	t/*χ* ^2^	*p* Value
Hb, POD1 (g/L)	116.00 ± 11.74	113.20 ± 13.57	1.566	0.119
110 < Hb, *n* (%)	71 (63.4)	52 (58.4)	9.785	0.02
100 < Hb ≤ 110, *n* (%)	35 (31.2)	20 (22.5)		
90 < Hb ≤ 100, *n* (%)	5 (4.5)	14 (15.7)		
80 < Hb ≤ 90, *n* (%)	1 (0.9)	3 (3.4)		
Hb ≤ 80, *n* (%)	0	0		
Hb, POD3 (g/L)	105.31 ± 12.62	103.09 ± 14.31	1.169	0.244
110 < Hb, *n* (%)	38 (33.9)	22 (24.7)	6.029	0.197
100 < Hb ≤ 110, *n* (%)	34 (30.4)	24 (27.0)		
90 < Hb ≤ 100, *n* (%)	26 (23.2)	32 (36.0)		
80 < Hb ≤ 90, *n* (%)	11 (9.8)	6 (6.7)		
Hb ≤ 80, *n* (%)	3 (2.7)	5 (5.6)		
△Hb (g/L)				
POD1 (g/L)	10.19 ± 7.30	13.69 ± 8.74	−3.027	0.003
POD3 (g/L)	20.88 ± 11.27	23.80 ± 10.15	−1.908	0.058
POD3‐POD1 (g/L)	10.69 ± 7.82	10.11 ± 6.82	0.548	0.585
TBL (mL)				
POD1 (mL)	311.33 ± 197.23	395.27 ± 252.05	−2.649	0.009
POD3 (mL)	606.81 ± 310.99	685.09 ± 302.39	−1.794	0.074
POD3‐POD1 (mL)	295.48 ± 224.13	289.83 ± 206.17	0.184	0.854

Abbreviations: △Hb, decline in haemoglobin level; N/A, not applicable; POD, postoperative day; pre‐OP, preoperation; TBL, total blood loss.

### Postoperative coagulation status

To further identify the influence of coagulation status on postoperative blood loss and ecchymosis formation, we analyzed postoperative coagulation status. On POD1, no statistically significant difference was found in terms of platelet count and coagulation profile between ecchymosis and nonecchymosis groups (Table 6). In TEG analysis, the ecchymosis group exhibited significantly higher K values (*p* = 0.032), and lower CI values (*p* = 0.025) compared with the nonecchymosis group. The subgroup analysis of CI showed that the proportion of patients with normal CI values (−3 to 3) was significantly more significant in the ecchymosis group than in the nonecchymosis group (*p* = 0.031), while the proportion of patients with high CI values (>3) was in the nonecchymosis group was higher than that in ecchymosis group (*p *= 0.024) (Table 6). As a comprehensive coagulation indicator, the difference distribution in CI values indicates that patients in the nonecchymosis group are in a relatively hypercoagulable state compared with those in the ecchymosis group.

**Table 6:** Postoperative coagulogram and TEG parameters in the ecchymosis and nonecchymosis groups.

Variables	Nonecchymosis (*n* = 112)	Ecchymosis (*n* = 89)	t or *χ* ^2^	*p* Value
APTT (s)	33.46 ± 3.83	32.60 ± 4.02	1.546	0.124
PT (s)	13.41 ± 1.20	13.44 ± 1.05	−0.151	0.88
TT (s)	16.70 ± 1.16	16.78 ± 1.37	−0.449	0.654
INR	1.03 ± 0.11	1.03 ± 0.09	−0.001	0.999
PTA (%)	97.40 ± 11.78	96.88 ± 13.31	0.296	0.768
PTR	1.03 ± 0.08	1.03 ± 0.07	−0.318	0.751
Platelet (10^9^/L)	196.68 ± 53.19	196.01 ± 45.61	0.094	0.925
R (min)	5.74 ± 1.41	6.08 ± 1.44	−1.708	0.089
K (min)	1.38 ± 0.41	1.52 ± 0.48	−2.155	0.032
α‐angle (°)	67.86 ± 10.53	66.52 ± 9.23	0.950	0.343
MA (mm)	63.63 ± 9.72	63.55 ± 4.29	0.076	0.939
CI	1.10 ± 1.70	0.55 ± 1.70	2.26	0.025
CI < −3, *n* (%)	0	1 (1.1)	7.186	0.028
−3 ≤ CI ≤ 3, *n* (%)	95 (84.8)	84 (94.4)		
3 < CI, *n* (%)	17 (15.2)	4 (4.5)		
LY30 (%)	0.5 (0.1, 1.7)	0.8 (0.1, 1.4)	−0.109	0.914
LY30 ≤ 3%, *n* (%)	100 (89.3)	85 (95.5)	2.619	0.106
3% < LY30, *n* (%)	12 (10.7)	4 (4.5)		

Abbreviations: APPT, activated partial thromboplastin time; CI, calculated coagulation index; EPL, estimated percent lysis; INR, international normalized ratio; K, K time; LY30, percentage of clot which has lysed after 30 min; MA, maximum amplitude; PTA, prothrombin time activity; PT, prothrombin time; PTR, posttransfusion refractoriness; R, reaction time; TEG, thromboelastography; TT, thrombin time.

**Figure 3:**
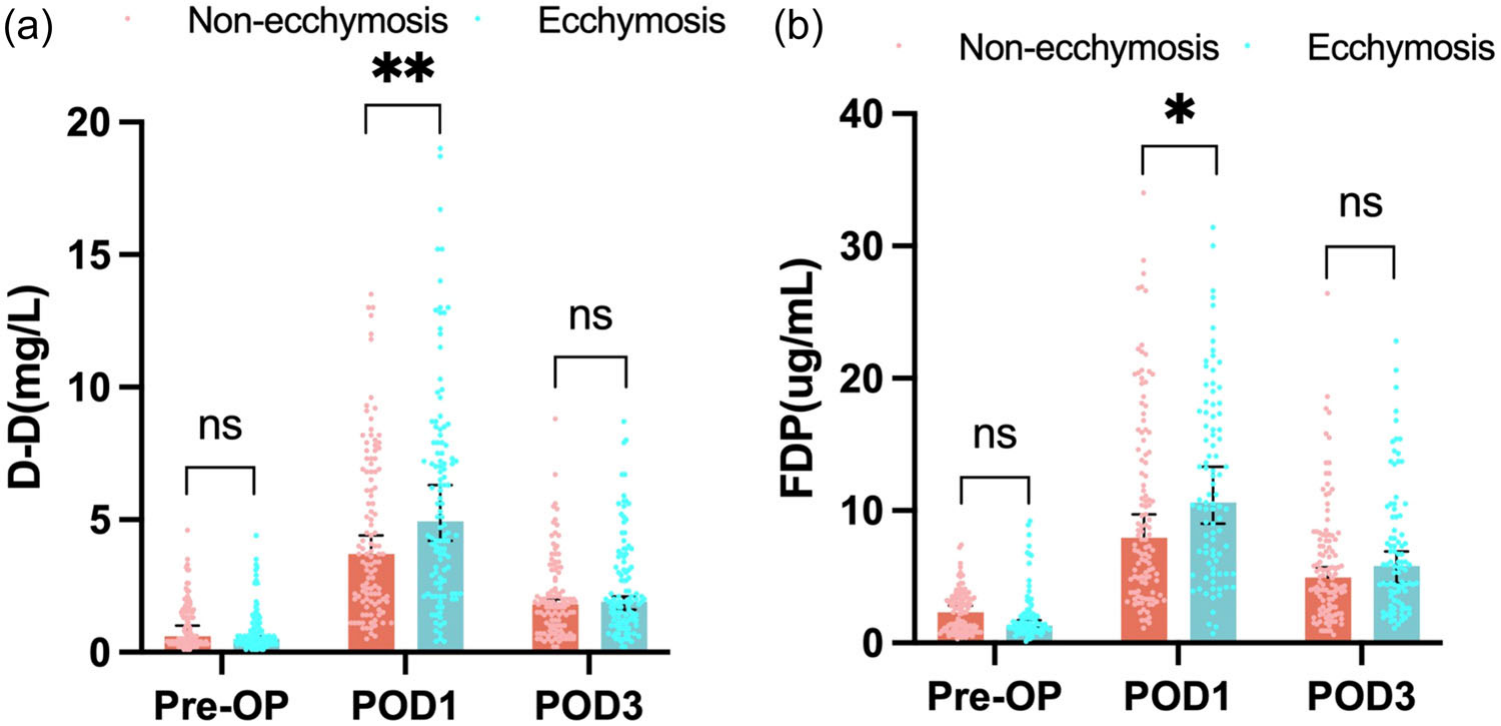
D‐dimer (a) and fibrinogen degradation products (b) changes on postoperative Day 1 and postoperative Day 3, respectively. *p < 0.05, **p < 0.01, ns, not significant.

As the other parameters for bleeding and VTE, fibrinolytic function is also closely associated with postoperative bleeding. Although LY30 distribution postoperatively did not show statistical significance, D‐dimer and fibrinogen degradation products values increased dramatically in the ecchymosis group compared with the nonecchymosis group on POD1 (*p* = 0.013 and 0.003), while this difference in existence on POD3 (*p *= 0.09 and 0.084) (Figure 3). These results indicated that patients with ecchymosis possess a relatively hyperfibrinolysis state compared with the nonecchymosis group on POD1, and this tendency disappeared with delayed anticoagulation treatment on POD3.

### Perioperative complications

Neither group of patients experienced severe systemic complications during the perioperative period (Table 7), including PE, postoperative sepsis, septic shock, cerebrovascular accident, acute renal failure and so on. The incidence of other complications was also not significantly different between the two groups (Table 7).

**Table 7:** Perioperative complications between ecchymosis and nonecchymosis groups.

Variables	Nonecchymosis (*n* = 112)	Ecchymosis (*n* = 89)	*χ* ^2^	*p* Value
Major systemic complications (no. [%])				
Death within POD90	0	0	N/A	N/A
PE	0	0	N/A	N/A
Postoperative sepsis	0	0	N/A	N/A
Septic shock	0	0	N/A	N/A
Cerebrovascular accident	0	0	N/A	N/A
Acute renal failure	0	0	N/A	N/A
Minor systemic complications (no. [%])				
IVT	5 (4.5)	4 (4.5)	0.001	0.992
DVT	1 (0.9)	1 (1.1)	0.027	0.87
Urinary tract infection	3 (2.7)	2 (2.2)	0.038	0.845
Pneumonia	1 (0.9)	0	0.799	0.372
Renal insufficiency	0	0	N/A	N/A
Major local complications (no. [%])				
Deep wound infection	1 (0.9)	0	0.799	0.372
Peripheral nerve injury	0	1 (1.1)	1.125	0.261
Minor local complications (no. [%])				
Superficial wound infection	1 (0.9)	2 (2.2)	0.619	0.432
Wound dehiscence	2 (1.8)	3 (3.4)	0.514	0.474

Abbreviations: DVT, deep vein thrombosis; IVT, intramuscular vein thrombosis; N/A, not applicable; PE, pulmonary embolism; POD, postoperative day; VTE, venous thromboembolism.

## DISCUSSION

Ecchymosis is still one of the most common complications following TKA. It not only impacts postoperative rehabilitation but is also an indicator of postoperative bleeding [2, 15, 32]. In this study, we proposed that postoperative ecchymosis indicates a hypocoagulable state and that delaying anticoagulation therapy by 1–2 days could be beneficial. The results suggested that delayed anticoagulation helped prevent exacerbation of post‐TKA blood loss without increasing postoperative complication rates. This outcome may be associated with the different postoperative coagulation and fibrinolysis states observed in the ecchymosis and nonecchymosis groups.

As a defined minor bleeding event related to postoperative coagulopathy, ecchymosis has been characterized as closely associated with hypocoagulation status [28]. Since excessive anticoagulation could increase the incidence of bleeding, wound complications and even excessive ecchymosis and blood loss, insufficiency anticoagulation could increase the risk of VTE [2, 6, 26]. Therefore, personalized anticoagulation therapy was suggested [4, 18, 28]. However, there is still controversy regarding whether to stop routinely used anticoagulant therapy in ecchymosis patients. In this study, we found that post‐TKA hypocoagulation and hyperfibrinolysis status in ecchymosis patients mainly occurred on POD1 and recovered on POD3 without anticoagulation therapy. Hence, we suggest delaying routinely used anticoagulant therapy for 1–2 days until the recovery of hypocoagulation and hyperfibrinolysis status for postoperative ecchymosis patients.

The perturbation of the coagulation and fibrinolytic systems of patients undergoing TKA was associated with postoperative VTE and bleeding. Hypercoagulation is positively associated with an increased risk of VTE, so routine anticoagulation after TKA is strongly recommended, as it dramatically reduces the incidence of VTE from 40% to 60% to <1% [7, 30]. On the other hand, hypocoagulation and hyperfibrinolysis are associated with postoperative bleeding, indicating that early and excessive anticoagulation therapy would increase the risk of postoperative bleeding. As coagulation, haemostatic function and fibrinolytic function are in a dynamic equilibrium, any alterations could lead to a pathological state or occurrence of postoperative complications [3, 5, 16]. Wang et al. [25] analyzed the changes in coagulation and fibrinolytic biomarkers after TKA and identified that the presence of hypercoagulation and hyperfibrinolysis increased the risk of VTE. However, Cote et al. [4] found that the symptomatic PE rate after TKA was relatively constant even when patients received potent anticoagulation, which may indicate the necessity of personalized anticoagulation treatment. In the present study, instead of only analyzing coagulation and fibrinolytic biomarkers, we take ecchymosis as the ‘barometer’ of thrombosis and bleeding. The results suggested a relatively hypocoagulation and hyperfibrinolysis state in the ecchymosis group compared with the nonecchymosis group, which applied theory evidence for delayed anticoagulation treatment for postoperative ecchymosis patients. As the hypocoagulation and hyperfibrinolysis state in the ecchymosis group recovered to the level of that in the nonecchymosis group, hence, we suggest postponing anticoagulation therapy for 1–2 days. Meanwhile, the use of the antifibrinolytic drug TXA is a potent strategy for the prevention and augmentation of post‐TKA ecchymosis [8, 21, 29].

As an indicator of postoperative bleeding, early recognition of ecchymosis is essential for personalized anticoagulation therapy. As shown in Figure 1, postoperative ecchymosis on POD1 typically manifests as a deepened colour of the skin, which usually might be neglected and dramatic ecchymosis is usually seen on POD2 or later. The hyperfibrinolysis following injury or surgery is associated with increased bleeding, primarily occurring within the initial hours postinjury [10], so we usually start anticoagulation therapy 12 h after TKA. Here, we found a relative hypofibrinolytic state in the ecchymosis group compared with the nonecchymosis group, which may be caused by delayed recovery of hyperfibrinolysis. Simultaneously, we found that postoperative hypocoagulation and hyperfibrinolysis state persist for 1–2 days, which indicates that discontinuing the use of anticoagulation therapy on POD3 or later may not be beneficial for preventing augmentation of ecchymosis or excessive bleeding. Therefore, characterizing risk factors for post‐TKA ecchymosis and dynamic monitoring coagulation and fibrinolytic biomarkers are necessary for high‐risk patients [2, 26, 28, 31].

This study has some limitations. First, it is a retrospective study with patients sourced from the same medical institution. However, strict inclusion and exclusion criteria were applied to minimize bias. Future research could be designed as a prospective study with larger sample sizes to provide support and validation. Second, the TBL was calculated using a widely used formula based on haematocrit levels, which can be influenced by perioperative fluid management strategies. Nonetheless, all enroled patients received the same perioperative fluid regimen upon admission, which should mitigate the impact on intergroup comparisons.

## CONCLUSION

Ecchymosis after TKA is a common complication. Patients with ecchymosis exhibited a relatively hypocoagulable and hyperfibrinolytic state with a stronger tendency for postoperative bleeding. Delayed anticoagulation in ecchymosis patients could effectively prevent further exacerbation of postoperative bleeding by avoiding a sustained hypocoagulable and hyperfibrinolytic state. Taking postoperative ecchymosis as a barometer for personalized delayed anticoagulation therapy could be beneficial in preventing and reducing excessive postoperative bleeding without increasing the risk of VTE.

## AUTHOR CONTRIBUTIONS

Xuefeng Luo and Dehua Wang conceived the study. Junyi Liao and Wei Huang designed the study. Xuefeng Luo, Dehua Wang, Junyi Liao, Wei Xu, Xi Liang and Wei Huang analyzed the data. Junyi Liao and Xuefeng Luo drafted the manuscript. All authors approved the final manuscript of this study.

## CONFLICT OF INTEREST STATEMENT

The authors declare no conflict of interest.

## ETHICS STATEMENT

All procedures performed in studies involving human participants were in accordance with the ethical standards of the national research committee and with the 1964 Helsinki Declaration and its later amendments or comparable ethical standards. This study has been approved by the Ethics Committee of The First Affiliated Hospital of Chongqing Medical University (ethical approval No. K2023‐636). All patients were provided written informed consent before participation.

## Data Availability

Data will be made available on reasonable request.
